# Indian superbug: analysis of laypress reports

**DOI:** 10.1186/1753-6561-5-S6-P301

**Published:** 2011-06-29

**Authors:** T Jeyaseelan Senthinath, P Revathi, R Vigneswari

**Affiliations:** 1 Microbiology, Chennai Medical College Hospital& Research Centre, Trichy, India; 2 Pharmacology, Chennai Medical College Hospital& Research Centre, Trichy, India

## Introduction / objectives

Following the publication on New Delhi Metalo β Lactamase (NDM) in Lancet, Indian lay press highlighted various issues related to it, during Aug-2010. The present report aim to provide the information disseminated in the press.

## Methods

Lay press materials related to Indian superbug and NDM published in 2 leading newspapers were collected daily. Consensus of 3 of the 5 was considered for categorisation. Simple descriptive statistics was used for analysis.

## Results

In the Newspaper The Hindu and New Indian Express, 19 and 10 items were disseminated respectively. The matters were educative remarks of professionals, community response, political interactions, pharmaceutical comments, statements of policy makers and economical aspects including Medical Tourism.

## Conclusion

The lay press reports brought out the existing suboptimal status of infection control policy, misuse of antimicrobials in India and the need for surveillances. Despite extensive press reports, practitioners & prescribers were neither bothered nor did changed their attitude.

## Disclosure of interest

None declared.

